# A genome-wide multiphenotypic association analysis identified candidate genes and gene ontology shared by four common risky behaviors

**DOI:** 10.18632/aging.102812

**Published:** 2020-02-22

**Authors:** Jing Ye, Li Liu, Xiaoqiao Xu, Yan Wen, Ping Li, Bolun Cheng, Shiqiang Cheng, Lu Zhang, Mei Ma, Xin Qi, Chujun Liang, Om Prakash Kafle, Cuiyan Wu, Sen Wang, Xi Wang, Yujie Ning, Xiaomeng Chu, Lin Niu, Feng Zhang

**Affiliations:** 1 Key Laboratory of Trace Elements and Endemic Diseases of National Health and Family Planning Commission, School of Public Health, Health Science Center, Xi’an Jiaotong University, Xi’an, China; 2 Clinical Research Center of Shaanxi Province for Dental and Maxillofacial Diseases, College of Stomatology, Xi’an Jiaotong University, Xi’an, China

**Keywords:** risky behaviors, regulatory SNP (rSNP), methylation quantitative trait loci (MeQTL), multivariate adaptive shrinkage (MASH)

## Abstract

Background: Risky behaviors can lead to huge economic and health losses. However, limited efforts are paid to explore the genetic mechanisms of risky behaviors.

Result: MASH analysis identified a group of target genes for risky behaviors, such as APBB2, MAPT and DCC. For GO enrichment analysis, FUMA detected multiple risky behaviors related GO terms and brain related diseases, such as regulation of neuron differentiation (adjusted *P* value = 2.84×10^-5^), autism spectrum disorder (adjusted *P* value =1.81×10^-27^) and intelligence (adjusted *P* value =5.89×10^-15^).

Conclusion: We reported multiple candidate genes and GO terms shared by the four risky behaviors, providing novel clues for understanding the genetic mechanism of risky behaviors.

Methods: Multivariate Adaptive Shrinkage (MASH) analysis was first applied to the GWAS data of four specific risky behaviors (automobile speeding, drinks per week, ever-smoker, number of sexual partners) to detect the common genetic variants shared by the four risky behaviors. Utilizing genomic functional annotation data of SNPs, the SNPs detected by MASH were then mapped to target genes. Finally, gene set enrichment analysis of the identified candidate genes were conducted by the FUMA platform to obtain risky behaviors related gene ontology (GO) terms as well as diseases and traits, respectively.

## INTRODUCTION

Risky behavior or risk-taking behavior has been defined as either a socially unacceptable volitional behavior with a potentially negative outcome in which precautions are not taken (e.g. speeding, drinking and driving), or a socially accepted behavior in which the danger is recognized (e.g. competitive sports and skydiving) [1]. It is well reported that risky behaviors were associated with high prevalence, low productivity and more generally with a decline of individual and collective well-being in the short, medium and long run [2], as well as high premature death and increased health care spending [3], leading to huge economic and health losses of society, especially automobile speeding, drinking, smoking, and multiple sexual partners. For example, a large US representative cohort over a period of 15 years follow-up shown a significant linear relationship for females and males under 60 years of age at baseline relationship between alcohol consumption and all-cause mortality [4].

Risky behaviors are a multifactorial disease with severe health and social consequences. Interestingly, past studies showed that risky behaviors were clinically recognized as a feature of several psychiatric disorders, including Attention deficit and hyperactivity disorder (ADHD) [5], Novelty seeking (NS) [6]. Moreover, the relationship between brain disorders and risky behaviors can also be explained from the genetic domain. Multiple studies pointed out gene expression in the brain region, especially in the prefrontal cortex, basal ganglia and midbrain, was associated with risky behaviors [7]. Therefore, clarifying the genetic mechanism of risky behaviors has the potential to inform the biology of complex psychiatric disorders.

Apart from the environmental effects, there are indeed genetic factors affecting the risky behaviors. It is worth emphasizing that a latest research found that genetic correlations for risky behaviors were significantly higher than other phenotypes, and that many lead SNPs were shared among their phenotypes [8]. Overlapping genetic factors usually imply a sharing of possible etiological mechanisms, so integrating multiple risk traits could offer new insights for us. Cortes et al. clustered the genetic risk profiles of 3,025 genome-wide independent loci of 19,155 disease classification codes from 320,644 participants in the UK Biobank, and identified 339 distinct disease association profiles, and used multiple methods to association the clusters to potential biological pathways [9]. In addition, limited efforts were paid to explore the common genetic factors shared by various risky behaviors. Therefore, joint analysis of multiple risky behaviors has the potential to provide insight into the biological mechanism of risky behaviors.

In recent years, GWAS has identified many genetic variants associated with complex diseases and traits [10, 11]. A large part of significant loci identified by GWAS were in non-coding chromosomal regions [12]. Interestingly, previous studies demonstrated the important roles of regulatory genetic variants in the pathogenesis of complex diseases and traits, such as the expression quantitative trait locus (eQTLs) and methylation quantitative trait locus (meQTL) [13–17]. For instance, Huan et al. integrated whole blood meQTL and GWAS with disease-related variants, and illuminated the pathways for cardiovascular disease [17]. Through the comprehensive functional annotation of schizophrenia susceptibility SNPs identified by GWAS, including eQTL and meQTL analysis, Niu et al. identified 447 target protein-coding genes [18]. Additionally, significant overlap between the SNPs identified by GWAS and regulatory genetic regions [19], functional SNPs located in protein-coding genes and non-coding genes, known as regulatory single nucleotide polymorphisms (rSNPs), playing a major and indirect role in regulating gene function [20]. Integrating GWAS data with rSNP and MeQTL had the potential to discover novel susceptibility genetic variants for human complex diseases [21, 22].

In this study, our goal was to explore the potential genetic factors shared by the four risky behaviors, especially those related to mental or psychological disorders. Accordingly, we first used MASH analysis to detect the common shared variants for the four common risky behaviors. Then the identified SNPs were mapped to target genes according to the genomic functional annotation data of eQTLs, MeQTLs and the SNPs near to known genes. Moreover, gene enrichment analysis was conducted to identify the significant pathways for risky behaviors.

## RESULTS

### Single gene analysis

For rSNP, we identified 4378 SNPs shared by the four risky behaviors, corresponding to 792 target regulatory genes, such as PLEKHM1, MAPT, APBB2, TFEC and KANSL1-AS1 (Supplementary Table 1). For MeQTL, 793 significant SNPs were detected, corresponding to 162 target genes, such as ADH1B, ADH1C, DCC, ARHGAP10 and ARHGAP27 (Supplementary Table 2). For the SNPs near to known genes, we detected 414 SNPs, corresponding to 89 target genes, such as PHC2, HYI, PTPRF, ELAVL4, and LRP8 (Supplementary Table 3).

### Functional gene sets enrichment analysis

For rSNP, we detected 56 candidate GO terms, such as Gene silencing (adjusted P value=5.73×10-12), Negative regulation of gene expression (adjusted P=5.73×10-12) and Chromatin silencing (adjusted P=1.13×10-11). For MeQTL, we identified 79 candidate GO terms, such as Neurogenesis (adjusted P=9.84×10-3), Subpallium development (adjusted P=9.84×10-3) and Striatum development (adjusted P=2.17×10-2). For the SNPs mapping to known genes, 215 candidate GO terms were identified, such as Regulation of collateral sprouting (adjusted P=2.84×10-5), Regulation of extent of cell growth (adjusted P=2.84×10-5) and Regulation of neuron differentiation (adjusted P=2.84×10-5). Moreover, FUMA analysis also detected multiple brain related diseases or traits enriched in the identified target genes of the four risky behaviors, such as Autism spectrum disorder (adjusted P value=1.81×10-27), Neuroticism (adjusted P=2.47×10-12) and Intelligence (adjusted P=5.89×10-15). More additional details were provided in Tables 1–3 and Supplementary Tables 4–6.

**Table 1:** Gene set enrichment analysis results of rSNP related genes associated with the four risky behaviors.

	**Gene Set**	**adjusted *P***
**Gene ontology enrichment analysis**	GO_GENE_SILENCING	5.73×10^-12^
	GO_NEGATIVE_REGULATION_OF_GENE_EXPRESSION_EPIGENETIC	5.73×10^-12^
	GO_CHROMATIN_SILENCING	1.13×10^-11^
	GO_CHROMATIN_ORGANIZATION	1.69×10^-08^
	GO_REGULATION_OF_GENE_EXPRESSION_EPIGENETIC	1.75×10^-08^
	GO_CHROMOSOME_ORGANIZATION	5.07×10^-08^
	GO_CHROMATIN_ASSEMBLY_OR_DISASSEMBLY	7.10×10^-07^
	GO_DNA_PACKAGING	7.10×10^-07^
	GO_PROTEIN_DNA_COMPLEX_SUBUNIT_ORGANIZATION	3.81×10^-06^
	GO_CHROMATIN_SILENCING_AT_RDNA	1.13×10^-04^
**Disease related gene set enrichment analysis**	Autism spectrum disorder or schizophrenia	1.81×10^-27^
	Alcohol use disorder (total score)	1.44×10^-12^
	Neuroticism	2.47×10^-12^
	General cognitive ability	2.47×10^-12^
	Intelligence (MTAG)	8.93×10^-09^
	Tacrolimus trough concentration in kidney transplant patients	1.22×10^-08^
	Facial emotion recognition (sad faces)	4.90×10^-07^
	Squamous cell lung carcinoma	3.10×10^-06^
	Cannabis use	3.28×10^-06^
	Lung cancer in ever smokers	4.03×10^-06^

**Table 2:** Gene set enrichment analysis results of MeQTL related genes associated with the four risky behaviors.

	**Gene Set**	**adjusted *P***
**Gene ontology enrichment analysis**	GO_NEUROGENESIS	9.84×10^-03^
	GO_SUBPALLIUM_DEVELOPMENT	9.84×10^-03^
	GO_HEAD_DEVELOPMENT	9.84×10^-03^
	GO_CENTRAL_NERVOUS_SYSTEM_NEURON_DIFFERENTIATION	9.84×10^-03^
	GO_NEURON_DIFFERENTIATION	9.84×10^-03^
	GO_REGULATION_OF_NEURON_DIFFERENTIATION	9.84×10^-03^
	GO_ENDOCRINE_SYSTEM_DEVELOPMENT	9.84×10^-03^
	GO_REGULATION_OF_VOLTAGE_GATED_CALCIUM_CHANNEL_ACTIVITY	1.06×10^-02^
	GO_REGULATION_OF_NERVOUS_SYSTEM_DEVELOPMENT	1.13×10^-02^
	GO_DEVELOPMENTAL_INDUCTION	1.13×10^-02^
**Disease related gene set enrichment analysis**	General cognitive ability	2.23×10^-18^
	Intelligence (MTAG)	5.89×10^-15^
	Self-reported risk-taking behaviour	8.24×10^-13^
	Alcohol use disorder (total score)	5.11×10^-11^
	Hand grip strength	2.58×10^-10^
	Educational attainment	1.01×10^-08^
	Alcohol consumption in current drinkers	4.72×10^-08^
	Body mass index	1.24×10^-07^
	Cognitive ability (MTAG)	1.50×10^-07^
	Alcohol use disorder (consumption score)	1.96×10^-07^

**Table 3:** Gene set enrichment analysis results of genes with SNPs showing association with the four risky behaviors.

	**Gene Set**	**adjusted *P***
**Gene ontology enrichment analysis**	GO_REGULATION_OF_COLLATERAL_SPROUTING	2.84×10^-05^
	GO_REGULATION_OF_EXTENT_OF_CELL_GROWTH	2.84×10^-05^
	GO_REGULATION_OF_NEURON_DIFFERENTIATION	2.84×10^-05^
	GO_NEGATIVE_REGULATION_OF_NITROGEN_COMPOUND_METABOLIC_PROCESS	2.84×10^-05^
	GO_REGULATION_OF_NERVOUS_SYSTEM_DEVELOPMENT	2.84×10^-05^
	GO_REGULATION_OF_CELL_DEVELOPMENT	1.03×10^-04^
	GO_NEUROGENESIS	1.03×10^-04^
	GO_NEGATIVE_REGULATION_OF_PROTEIN_OLIGOMERIZATION	1.03×10^-04^
	GO_REGULATION_OF_NEURON_PROJECTION_DEVELOPMENT	1.19×10^-04^
	GO_ETHANOL_METABOLIC_PROCESS	1.19×10^-04^
**Disease related gene set enrichment analysis**	Alcohol use disorder (total score)	2.77×10^-08^
	Cognitive ability (MTAG)	2.77×10^-08^
	General cognitive ability	2.77×10^-08^
	Alcohol use disorder (consumption score)	7.78×10^-08^
	Intelligence (MTAG)	3.27×10^-07^
	Alzheimer's disease in APOE e4- carriers	3.11×10^-06^
	Cognitive ability	3.17×10^-06^
	Hand grip strength	4.46×10^-06^
	Parental longevity (father's attained age)	5.35×10^-06^
	Autism spectrum disorder or schizophrenia	1.35×10^-05^

## DISCUSSION

Keller and Cannon’s watershed analogy of the genotype–phenotype relationship that “the more upstream the phenotype is, the closer its relationship to the genetic variants that affect it [23, 24]. Thereby, we conducted a large-scale genome-wide multiphenotypic integrative analysis for four common risky behaviors. We reported multiple candidate genes and GO terms shared by the four risky behaviors. Our study had the potential to explore the genetic mechanism underlying risky behaviors and further elucidate the biological mechanisms of the development of mental disorders.

Multiphenotypic analysis identified several common genes associated with four risky behaviors, such as APBB2, MAPT and DCC. APBB2, the protein encoded by this gene interacts with the cytoplasmic domains of amyloid beta (A4) precursor protein and amyloid beta (A4) precursor-like protein 2. This protein contains two phosphotyrosine binding (PTB) domains that are thought to play a role in signal transduction [25]. Polymorphisms in this gene have been associated with Alzheimer's disease [26]. Another interesting new finding was that they used PCR-RFLP to analyze the substitution of hCV1558625 (rs13133980) and rs13133980, and discovered that hcv1558625-rs13133980 AG haplotype increased the relative risk of severe cognitive impairment in centenarians [27]. In addition, the APBB2 rs13133980 G allele was more highly expressed in centenarians with severe cognitive impairment than in the individuals without cognitive impairment [27].

MAPT, a microtubule-related protein, whose transcript undergoes complex, regulated selective splicing, producing a variety of mRNA species. MAPT transcription products are expressed differently in the nervous system, depending on the stage of neuron maturation and the type of neuron [25]. Mutations in the MAPT gene are associated with a variety of neurodegenerative diseases, such as Alzheimer's disease [26], frontotemporal dementia, and cortical basal cell degeneration [25]. By analyzing MAPT region expression, splicing and regulation in 2011 brain samples from 439 individuals, a survey found that regional differences in MAPT mRNA expression and splicing in human brain were highly correlated with the total expression level of tau protein [28]. Furthermore, they hypothesized that genetic risk factors for neurodegenerative disease at the MAPT site may play a role by altering mRNA splicing in different areas of the brain, rather than by the overall expression of the MAPT gene [28].

DCC encodes the netrin 1 receptor. Transmembrane proteins are members of the immunoglobulin superfamily of cell adhesion molecules that mediate neuronal growth cones and are the source of axon-directed netrin 1 ligands [25]. Variations in DCC may determine differential predisposition to mPFC disorders in humans, clarified by Manitt et al. [29] Their results found that DCC expression was elevated in the brains of antidepressant-free subjects who committed suicide. Horn K et al. [30] found that the dendritic spines of the pyramidal neurons of wild-type mice were rich in DCC, and then demonstrated that selective deletion of DCC in the brain neurons in the adult forebrain resulted in long-term potential loss (LTP), complete long-term depression, short dendritic spines, and impaired spatial and recognition memory, through the DCC knockout mice experiment.

GO enrichment analysis identified multiple significant GO terms, which were involved in the development of the brain and mental, such as neurogenesis, regulation of neuron differentiation and striatum development. Neurogenesis, or called neural cell differentiation, a process of producing functional neurons from adult neural precursors throughout life in a limited number of mammalian brain regions [31]. Deng et al. suggested that an important role for adult hippocampal neurogenes is learning and memory [32]. According to Kang et al.’s research, dysregulation in adult neurogenesis are implicated in psychiatric diseases in humans, such as affective disorders, schizophrenia, and drug addiction [33].

Regulation of neuron differentiation, which is defined as any process, modulates the frequency, rate or extent of neuron differentiation. A previous study combined the genome-wide disease risk profile of GWAS with the longitudinal in vitro gene expression profile of human neuronal differentiation using an analytical framework they developed to demonstrate that the cumulative impact of risk loci for specific psychiatric disorders is significantly correlated with genes that are differentially expressed during neuronal differentiation [34].

Striatum development refers to the process from initial formation to maturation of the striatum. The striatum is a region of the forebrain that consists of the caudate nucleus, putamen nucleus, and striatum base. Similarly, studies linked striatum development to mental illness, such as delayed development of the ventral striatum during adolescence reflects emotional neglect and predicts depressive symptoms [35].

Additionally, we detected multiple brain related diseases or traits enriched in the identified target genes of the four risky behaviors, such as SCZ and intelligence. SCZ is an idiopathic mental disorder with high heritability. Previous studies showed that schizophrenia is associated with neural calcium channels, which are one of the pathogenesis of bipolar disorder and autism [36]. Sullivan et al.'s study described the strong effects of eight rare copy number variants on schizophrenia, and these associations might also be associated with autism, mental retardation or epilepsy, which are usually not disease-specific [37]. In addition, analysis of the genetic characteristics found a significant relationship between intelligence and changes in the expression of the brain and pituitary gland. It also suggested that neurogenesis was the process by which new neurons are created, a process previously associated with human intelligence using GWAS data [38]. Interestingly, combined with our findings and previous research, it seemed that many psychiatric disorders also have codependent biological mechanisms that rely on similar gene regulation or expression, or are partly due to common genetic influence. Our study confirmed this finding and provided some assistance in further elucidating the biological mechanism of risky behavior.

One strengths of this study is the combination of multiple risky behaviors and integrating it with genomic functional annotation of regulatory genetic variants. Complex disorders or traits are usually regulated by gene expression, methylation, microRNAs, and epigenomics as a whole. Due to some limitation of GWAS method [12] and the important role of regulatory genetic variants [13–17, 19], integrating GWAS and regulatory genetic variants data can discover novel candidate genes for mental disorders in this study. Meanwhile, GWAS of common diseases have revealed a wide range of pleiotropic, resulting in significant genetic correlations among different traits [9]. For instance, Cross-disorder Group of the Psychiatric Genomics Consortium observed the genetic correlation among five mental disorders based on genome-wide SNPs data [39]. Overlapping genetic risks imply a sharing of possible etiological mechanisms. Ellinghaus et al. 's analysis of five chronic inflammatory diseases identified 27 new associations and highlighted disease-specific patterns in shared loci [40]. Furthermore, previous studies provided evidence that different risky behaviors occurred simultaneously with the same mental disease [5–7]. Therefore, joint analysis of multiple risky behaviors has the potential to provide insight into the biological mechanism of risky behaviors. Our study identified multiple risk behaviors associated genes, which have been suggested to be involved in the development of mental disorders, such as APBB2 [26] and DCC [30]. Further GO analysis identified multiple risk behavior related GO terms, functionally involved in brain development and mental disorders, such as neurogenesis, regulation of neuron differentiation and striatum development.

In our study, we used MASH analysis to explore the common genetic factors shared by the four common risky behaviors. MASH was developed based on recent methods [41, 42], and it combines the advantages of existing methods while overcomes the major limitations of them. MASH gained more power compared to a tissue-by-tissue analysis and ANOVA or simple linear regression. The most important feature of MASH is that it facilitates more estimation and assessment of effect-size heterogeneity than simple “shared/condition-specific” assessments [41]. In particular, MASH is generic and adaptive. It is generic in that it can take as input any matrix of Z scores (or, better, a matrix of effect estimates and their corresponding standard errors) testing many effects in many conditions. And MASH is adaptive in that it learns patterns of sharing of multivariate effects from the data, allowing it to maximize power and precision for each setting. Urbut et al. [41] conducted a detailed analysis of locally-acting (“cis”) eQTLs in 44 human tissues through MASH, and compared the performance of MASH with that of ASH (a univariate shrinkage procedure) [43] and BMATILE (a multivariate model) [42]. It turns out that MASH outperformed other methods, particularly in the shared and structured effects scenario. However, it should be noted that MASH does not distinguish the causal associations and those caused by linkage disequilibrium (LD). When jointly analyzing GWAS and eQTLs data, a SNP identified by GWAS may be a significant eQTL simply because it is in LD with another causal SNP.

Additionally, there are some limitations of this study. First, due to the inclusion criteria of risky behaviors from our GAWS data sources [8], we analyzed four common risk behaviors in this study. The four risky behaviors have been proved to be related to psychological or mental disorders [5–7, 44–46]. Furthermore, they have been demonstrated to be a powerful predictor for injury, risk tolerance and other problems [1, 3]. However, it is worthy to jointly analyze more risky behaviors in future studies. Second, our analysis only included the genetically regulated portion of gene expression. Therefore, it could not capture or interpret the variance of expression caused by environment factors, which may also contribute to development of psychiatric disorders. Third, we analyzed the genetic data from European cohorts in this study. Therefore, it should be carefully to apply our results to other populations.

In conclusion, multiple genes and GO terms shared by the four risky behaviors were reported in our study. And the results supported the functional relevance of brain development with risky behaviors from the genetic domain, which can offer some help to further elucidate the biological mechanisms of the relationship. Due to many mental disorders also had mutual biological mechanism reliant on the similar genetic regulation or expression, our results may do a lot help to construct better multi-gene scores to measure environmental, demographic, and genetic factors interacting or building neurological scores.

## MATERIALS AND METHODS

### GWAS data of risky behaviors

In this study, we analyzed four common risky behaviors, including automobile speeding, alcohol drinking, smoking, and multiple sexual partners. Automobile speeding, alcohol drinking, smoking, and multiple sexual partners are major common risky behaviors, which have been demonstrated to be a powerful predictor for injury, risk tolerance and other problems [2, 3]. The GAWS data of the four risky behaviors were obtained from the published study [8]. It consisted of over 1 million European-ancestry participants for the four risky behaviors, including 404,291 for automobile speeding propensity, 414,343 for drinks per week, 370,711 for number of sexual partners and 518,633 for smoker, respectively. Genotyping was performed by using a range of commercially available genotyping arrays, such as the UK BiLEVE array [47] and the UK Biobank Axiom array [48]. Extensive quality-control procedures were applied to the cohort-level summary statistics, including the EasyQC software developed by the GIANT consortium [49], and only SNPs with minor allele frequency (MAF) greater than 0.001 were analyzed. IMPUTE4 was applied for genotype imputation [48]. The top ten (or more) principal components of the genetic relatedness matrix, sex and birth year were controlled during the GWAS. At last, each behavior consisted of approximately 11,515,000 SNPs in this study. More detailed information about cohorts, inclusion criteria for risky behaviors, genotyping and imputation can be found in the published study [8].

### Multiple traits integrative analysis

The MASH analysis was applied to the GWAS datasets of the four risky behaviors to detect the SNPs associated with all of the four risky behaviors. MASH (https://github.com/stephenslab/mashr) can estimate and test multiple effects under multiple conditions. The approach improves on existing methods to allow arbitrary correlation of effect size between conditions and improves effect size assessment, which is helpful for more quantitative assessment of effect size heterogeneity [41]. The SNPs associated with the four risky behaviors were detected by MASH and then mapped to target genes according to the genomic annotation data of rSNP-target genes, MeQTL-target genes and the SNPs near to known genes, respectively. We used “canonical” type to set up covariance matrix and then to fit the model. The sharing important signals between each pair of conditions were selected. And the default definition for sharing from MASH software is "the same sign and within a factor 0.5 of each other”.

### Annotation data of regulatory SNPs, MeQTL and the SNPs mapping to near genes

The genomic annotation data of rSNPs was obtained from the rSNPBase 3.1 database (http://rsnp3.psych.ac.cn/). rSNPBase provides genomic annotation of SNP-related regulatory element-target gene pairs [50]. Currently, rSNPBase database contains nearly 119,630,196 rSNP annotation entries on SNP regulatory information. Genomic similarities or widely used reference databases were used to analyze functional associations between regulatory elements and target genes [51]. The MeQTLs–target gene annotation data were collected from published studies [52]. In short, about 4.5 million loci were measured in 697 subjects. SNPs were genotyped using Affymetrix genome-wide SNP Arrays 5.0 or 6.0 or Illumina OmniExpress. The Minimac method was used to input 1000 genomic reference versions of v3 with the lower MAF>0.05 and r2 >0.5 as thresholds. 4,761,800 SNPs were identified for MeQTL correlation after quality control. For mapping SNPs to near genes, a physical short of 500 kb was to link a SNP and a gene, because most enhancers and repressors are < 500 kb away from genes, and most linkage disequilibrium blocks are < 500 kb.

### Functional gene sets enrichment analysis

The identified candidate genes shared by the four risky behaviors were subjected to gene set enrichment analysis, implemented by the GENE2FUNC of the FUMA tool [20]. FUMA [20] is a platform for annotating, sorting, visualizing, and interpreting GWAS results. For every input gene, GENE2FUNC provides information about tissue specificity, the enrichment of publicly available gene sets, and the expression of different tissue types. The genes were tested for representation in different functional gene sets, including GO terms and various diseases or traits related gene sets. The Benjamini-Hochberg false discovery rate (FDR) was recommended by the FUMA software [20], and used for controlling the potential impact of multiple testing problem in this study. The adjusted *P* value cut-off was 0.05 and minimum overlapping genes with gene-sets was assigned 2 during the FUMA analysis. Additionally, the ensemble version v92 and GTEx v8 were chosen for FUMA analysis.

## Supplementary Material

Supplementary Table 1

Supplementary Table 2

Supplementary Table 3

Supplementary Table 4

Supplementary Table 5

Supplementary Table 6
